# Pyrrolidine-2,5-dione

**DOI:** 10.1107/S1600536812035672

**Published:** 2012-08-23

**Authors:** Min Yu, Xing Huang, Feng Gao

**Affiliations:** a Agronomy College, Sichuan Agricultural University, No. 211, Huiming Road, Wenjiang Region, Chengdu 611130, People’s Republic of China

## Abstract

In the title compound, C_4_H_5_NO_2_, the non-H atoms are nearly coplanar, with a maximum deviation of 0.030 (1) Å. In the crystal, pairs of mol­ecules are linked by N—H⋯O hydrogen bonds into inversion dimers.

## Related literature

For the synthesis, see: Ilieva *et al.* (2012 ▶); Adib *et al.* (2010 ▶). For the bioactivity of pyrrolidine-2,5-dione derivatives, see: Obniska *et al.* (2012 ▶); Ha *et al.* (2011 ▶); Kaminski *et al.* (2011 ▶). For related structures, see: Khorasani & Fernandes (2012 ▶); Mayes *et al.* (2008 ▶).
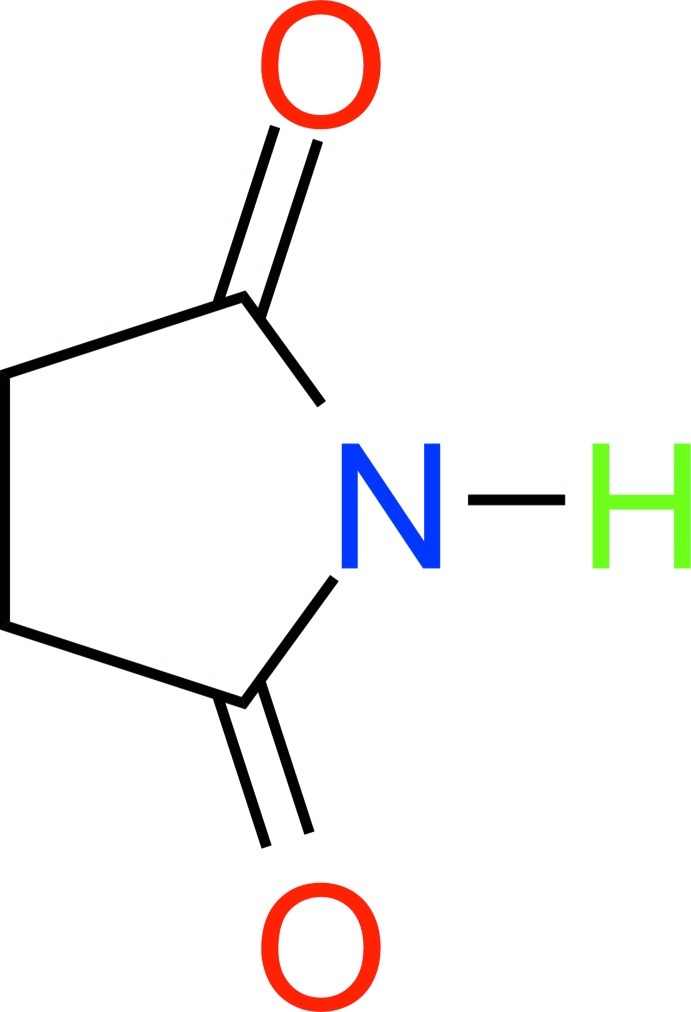


## Experimental

### 

#### Crystal data

C_4_H_5_NO_2_
*M* *_r_* = 99.09
Orthorhombic, 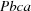

*a* = 7.3661 (4) Å
*b* = 9.5504 (5) Å
*c* = 12.8501 (7) Å
*V* = 904.00 (8) Å^3^
*Z* = 8
Mo *K*α radiation
μ = 0.12 mm^−1^
*T* = 135 K
0.40 × 0.35 × 0.30 mm

#### Data collection

Agilent Xcalibur Eos diffractometer
Absorption correction: multi-scan (*CrysAlis PRO*; Agilent, 2010 ▶) *T*_min_ = 0.911, *T*_max_ = 1.000
2022 measured reflections
915 independent reflections
732 reflections with *I* > 2σ(*I*)
*R*_int_ = 0.018

#### Refinement

*R*[*F* ^2^ > 2σ(*F* ^2^)] = 0.039
*wR*(*F* ^2^) = 0.097
*S* = 1.05
915 reflections
64 parameters
H-atom parameters constrained
Δρ_max_ = 0.15 e Å^−3^
Δρ_min_ = −0.29 e Å^−3^

### 

Data collection: *CrysAlis PRO* (Agilent, 2010 ▶); cell refinement: *CrysAlis PRO*; data reduction: *CrysAlis PRO*; program(s) used to solve structure: *SHELXTL* (Sheldrick, 2008 ▶); program(s) used to refine structure: *SHELXTL*; molecular graphics: *OLEX2* (Dolomanov *et al.*, 2009 ▶); software used to prepare material for publication: *publCIF* (Westrip, 2010 ▶).

## Supplementary Material

Crystal structure: contains datablock(s) I, global. DOI: 10.1107/S1600536812035672/xu5605sup1.cif

Structure factors: contains datablock(s) I. DOI: 10.1107/S1600536812035672/xu5605Isup2.hkl

Supplementary material file. DOI: 10.1107/S1600536812035672/xu5605Isup3.cml

Additional supplementary materials:  crystallographic information; 3D view; checkCIF report

## Figures and Tables

**Table 1:** Hydrogen-bond geometry (Å, °)

*D*—H⋯*A*	*D*—H	H⋯*A*	*D*⋯*A*	*D*—H⋯*A*
N1—H1⋯O2^i^	0.86	2.00	2.8548 (16)	176

Symmetry code: (i) 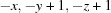.

**Fig. 1.**
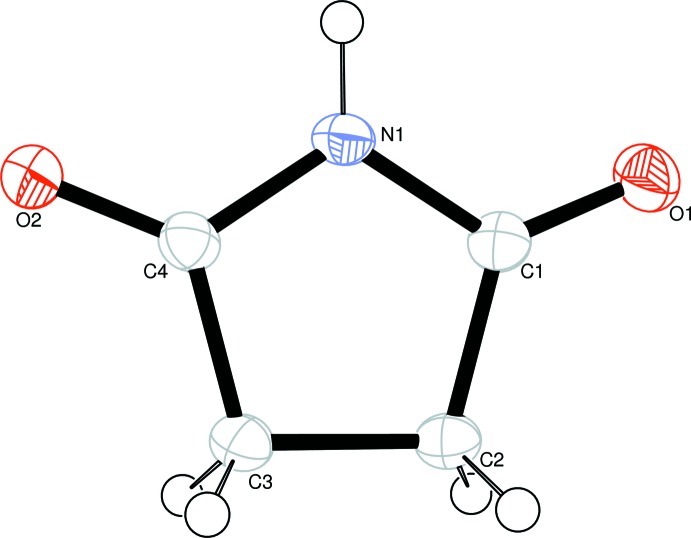
The title compound with displacement ellipsoids drawn at the 50% probability level. H atoms are shown as spheres of arbitary radius.

**Fig. 2.**
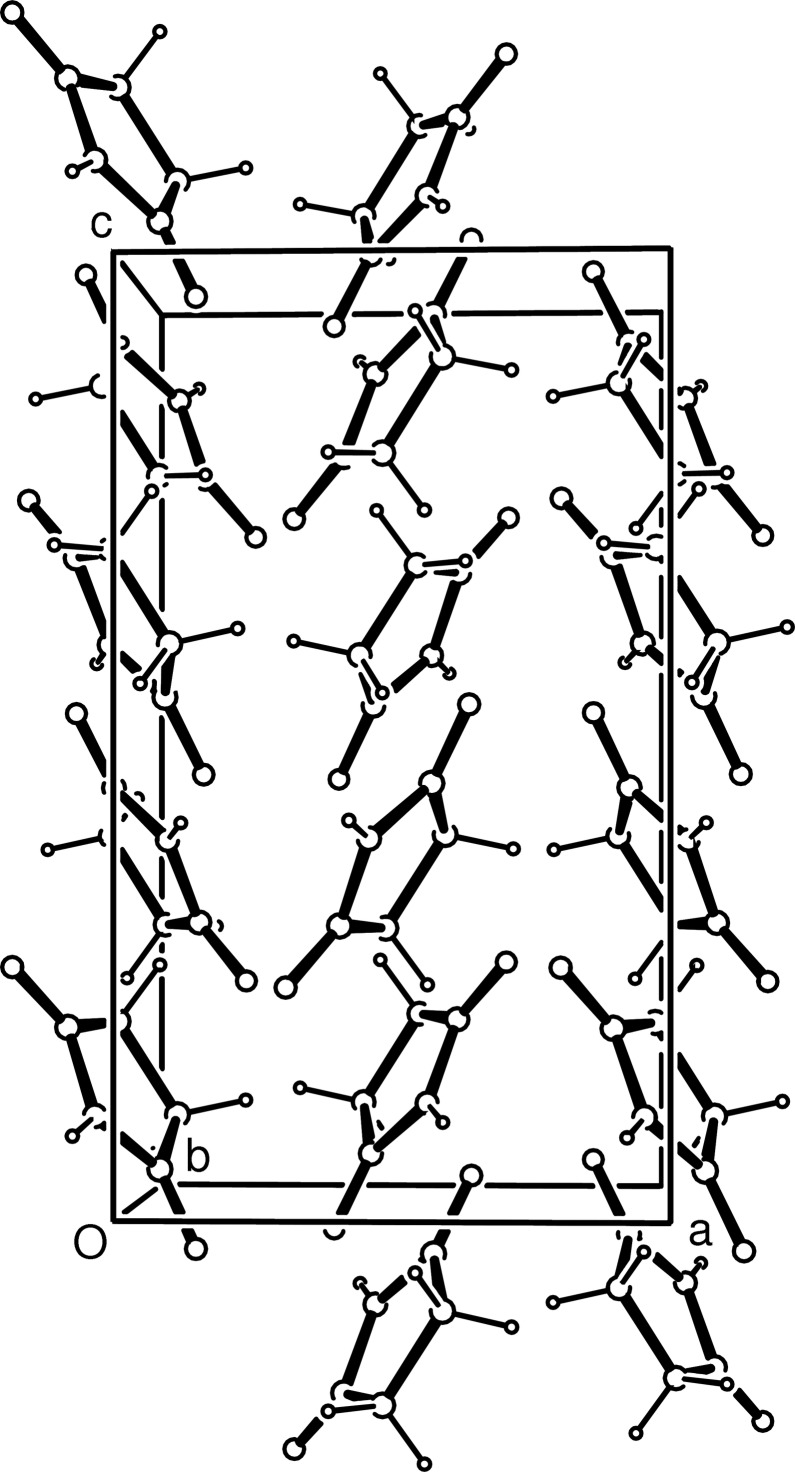
Plane-to-plane stacking of alternate molecules parallel to the α axis.

## supplementary crystallographic information

### Comment

Pyrrolidine-2,5-diones derivates are an important class of heterocylic compounds with essential applications in the organic synthesis and medicinal chemistry. In the organic field, pyrrolidine-2,5-diones derivates, such as well known 1-bromopyrrolidine-2,5-dione (NBS), are the most commonly used halogenation reagents. Meanwhile, pyrrolidine-2,5-diones derivates exhibit numerous bioactivity, especially in anticonvulsant (Obniska *et al.*, 2012; Kaminski *et al.*, 2011) and tyrosinase inhibitory activity (Ha *et al.*, 2011). Therefore, development of new and efficient strategies for the synthesis of multi-substituted pyrrolidine-2,5-diones is also the current hot in organic and medical chemistry (Ilieva *et al.*, 2012; Adib *et al.*. 2010). Several crystal structures of title compound derivates have been reported (Khorasani and Fernandes 2012; Mayes *et al.*, 2008), but crystal data of pyrrolidine-2,5-dione has not been investigated. Herein, we report the synthesis and completely crystal data of title compound.

The molecular structure of pyrrolidine-2,5-dione is shown in Fig. 1. The bond lengths and angles are within normal ranges. In the crystal, the molecules are connected through intermolecular N–H···O hydrogen bond (Table 1).

### Experimental

The single crystals of pyrrolidine-2,5-dione, C_4_H_5_NO_2_, were recrystallized from acetone at room temperature to give the desired crystals suitable for single-crystal X-ray diffraction, mounted inert oil and transferred to the cold gas stream of the diffractometer

### Refinement

C and N bound-H (atoms were included in idealized positions and refined using a riding-model approximation, with C—H bond lengths fixed at 1.00 Å, 0.99 Å, for methine and methylene H atoms respectively. *U*_iso_(H) values were fixed at 1.2Ueq of the parent atoms for all H atoms.

### Crystal data

**Table:**

C_4_H_5_NO_2_	*F*(000) = 416
*M**_r_* = 99.09	*D*_x_ = 1.456 Mg m^−^^3^
Orthorhombic, *P**b**c**a*	Mo *K*α radiation, λ = 0.7107 Å
Hall symbol: -P 2ac 2ab	Cell parameters from 806 reflections
*a* = 7.3661 (4) Å	θ = 3.2–28.9°
*b* = 9.5504 (5) Å	µ = 0.12 mm^−^^1^
*c* = 12.8501 (7) Å	*T* = 135 K
*V* = 904.00 (8) Å^3^	Block, colourless
*Z* = 8	0.40 × 0.35 × 0.30 mm

### Data collection

**Table:**

Agilent Xcalibur Eos diffractometer	915 independent reflections
Radiation source: Enhance (Mo) X-ray Source	732 reflections with *I* > 2σ(*I*)
Graphite monochromator	*R*_int_ = 0.018
Detector resolution: 16.0874 pixels mm^-1^	θ_max_ = 26.4°, θ_min_ = 3.2°
ω scans	*h* = −8→9
Absorption correction: multi-scan (*CrysAlis PRO*; Agilent, 2010)	*k* = −11→6
*T*_min_ = 0.911, *T*_max_ = 1.000	*l* = −16→14
2022 measured reflections	

### Refinement

**Table:**

Refinement on *F*^2^	Primary atom site location: structure-invariant direct methods
Least-squares matrix: full	Secondary atom site location: difference Fourier map
*R*[*F*^2^ > 2σ(*F*^2^)] = 0.039	Hydrogen site location: inferred from neighbouring sites
*wR*(*F*^2^) = 0.097	H-atom parameters constrained
*S* = 1.05	*w* = 1/[σ^2^(*F*_o_^2^) + (0.0457*P*)^2^ + 0.1774*P*] where *P* = (*F*_o_^2^ + 2*F*_c_^2^)/3
915 reflections	(Δ/σ)_max_ < 0.001
64 parameters	Δρ_max_ = 0.15 e Å^−^^3^
0 restraints	Δρ_min_ = −0.29 e Å^−^^3^

### Special details

**Table:**

Geometry. All e.s.d.'s (except the e.s.d. in the dihedral angle between two l.s. planes) are estimated using the full covariance matrix. The cell e.s.d.'s are taken into account individually in the estimation of e.s.d.'s in distances, angles and torsion angles; correlations between e.s.d.'s in cell parameters are only used when they are defined by crystal symmetry. An approximate (isotropic) treatment of cell e.s.d.'s is used for estimating e.s.d.'s involving l.s. planes.
Refinement. Refinement of *F*^2^ against ALL reflections. The weighted *R*-factor *wR* and goodness of fit *S* are based on *F*^2^, conventional *R*-factors *R* are based on *F*, with *F* set to zero for negative *F*^2^. The threshold expression of *F*^2^ > σ(*F*^2^) is used only for calculating *R*-factors(gt) *etc*. and is not relevant to the choice of reflections for refinement. *R*-factors based on *F*^2^ are statistically about twice as large as those based on *F*, and *R*- factors based on ALL data will be even larger.

### Fractional atomic coordinates and isotropic or equivalent isotropic displacement parameters (Å^2^)

**Table:**

	*x*	*y*	*z*	*U*_iso_*/*U*_eq_	
N1	0.04477 (16)	0.61457 (13)	0.39506 (9)	0.0197 (3)	
H1	0.0772	0.5317	0.4135	0.024*	
O1	0.19664 (16)	0.61963 (11)	0.23948 (8)	0.0291 (3)	
O2	−0.13630 (16)	0.66055 (12)	0.53572 (8)	0.0312 (3)	
C2	0.0226 (2)	0.82282 (15)	0.30007 (12)	0.0211 (4)	
H2A	−0.0546	0.8346	0.2395	0.025*	
H2B	0.1184	0.8924	0.2979	0.025*	
C1	0.10102 (19)	0.67648 (15)	0.30357 (12)	0.0197 (4)	
C4	−0.0668 (2)	0.69665 (16)	0.45340 (11)	0.0204 (4)	
C3	−0.0874 (2)	0.83593 (16)	0.40006 (11)	0.0230 (4)	
H3A	−0.0403	0.9108	0.4434	0.028*	
H3B	−0.2139	0.8549	0.3847	0.028*	

### Atomic displacement parameters (Å^2^)

**Table:**

	*U*^11^	*U*^22^	*U*^33^	*U*^12^	*U*^13^	*U*^23^
N1	0.0206 (6)	0.0160 (6)	0.0226 (7)	0.0026 (5)	−0.0004 (5)	0.0014 (5)
O1	0.0309 (6)	0.0239 (6)	0.0325 (6)	−0.0004 (5)	0.0126 (5)	−0.0002 (5)
O2	0.0430 (7)	0.0274 (6)	0.0232 (6)	0.0118 (6)	0.0087 (6)	0.0049 (5)
C2	0.0215 (7)	0.0171 (7)	0.0248 (8)	−0.0011 (6)	−0.0004 (6)	0.0020 (6)
C1	0.0173 (7)	0.0186 (8)	0.0231 (7)	−0.0044 (6)	0.0000 (6)	0.0002 (6)
C4	0.0221 (7)	0.0196 (7)	0.0194 (8)	0.0021 (6)	−0.0023 (6)	−0.0016 (6)
C3	0.0301 (8)	0.0167 (7)	0.0222 (8)	0.0015 (7)	0.0003 (7)	−0.0012 (6)

### Geometric parameters (Å, º)

**Table:**

N1—H1	0.8600	C2—H2B	0.9700
N1—C1	1.3796 (19)	C2—C1	1.513 (2)
N1—C4	1.3609 (19)	C2—C3	1.524 (2)
O1—C1	1.2121 (17)	C4—C3	1.504 (2)
O2—C4	1.2246 (18)	C3—H3A	0.9700
C2—H2A	0.9700	C3—H3B	0.9700
			
C1—N1—H1	123.1	O1—C1—C2	127.99 (14)
C4—N1—H1	123.1	N1—C4—C3	108.61 (12)
C4—N1—C1	113.83 (13)	O2—C4—N1	124.48 (14)
H2A—C2—H2B	108.9	O2—C4—C3	126.91 (14)
C1—C2—H2A	110.8	C2—C3—H3A	110.8
C1—C2—H2B	110.8	C2—C3—H3B	110.8
C1—C2—C3	104.70 (12)	C4—C3—C2	104.96 (12)
C3—C2—H2A	110.8	C4—C3—H3A	110.8
C3—C2—H2B	110.8	C4—C3—H3B	110.8
N1—C1—C2	107.85 (12)	H3A—C3—H3B	108.8
O1—C1—N1	124.16 (14)		
			
N1—C4—C3—C2	1.90 (16)	C4—N1—C1—O1	−177.92 (14)
O2—C4—C3—C2	−178.47 (14)	C4—N1—C1—C2	2.11 (16)
C1—N1—C4—O2	177.78 (14)	C3—C2—C1—N1	−0.75 (15)
C1—N1—C4—C3	−2.58 (17)	C3—C2—C1—O1	179.29 (15)
C1—C2—C3—C4	−0.66 (15)		

### Hydrogen-bond geometry (Å, º)

**Table:**

*D*—H···*A*	*D*—H	H···*A*	*D*···*A*	*D*—H···*A*
N1—H1···O2^i^	0.86	2.00	2.8548 (16)	176

Symmetry code: (i) −*x*, −*y*+1, −*z*+1.

## References

[bb1] Adib, M., Ansari, A., Fatemi, S., Bijanzadeh, H. R. & Zhu, L. G. (2010). *Tetrahedron*, **66**, 2723–2727.

[bb2] Agilent (2010). *CrysAlis PRO* Agilent Technologies, Yarnton, England.

[bb3] Dolomanov, O. V., Bourhis, L. J., Gildea, R. J., Howard, J. A. K. & Puschmann, H. (2009). *J. Appl. Cryst.* **42**, 339–341.

[bb4] Ha, Y. M., Kim, J., Parkl, Y. J., Park, D., Choi, Y. J., Kim, J. M., Chung, K. W., Han, Y. K., Park, J. Y., Lee, J. Y., Moon, H. R. & Chung, H. Y. (2011). *Med. Chem. Commun.* **2**, 542–549.

[bb5] Ilieva, E. D., Petkova, N. I. & Nikolova, R. D. (2012). *Molecules*, **17**, 4936–4949. 10.3390/molecules17054936 PMC6268139 22547316

[bb6] Kaminski, K., Rzepka, S. & Obniska, J. (2011). *Bioorg. Med. Chem. Lett.* **21**, 5800–5803. 10.1016/j.bmcl.2011.07.118 21875804

[bb7] Khorasani, S. & Fernandes, M. A. (2012). *Acta Cryst.* E**68**, o1503. 10.1107/S160053681201536X PMC3344612 22590374

[bb8] Mayes, B. A., McGarry, P., Moussa, A. & Watkin, D. J. (2008). *Acta Cryst.* E**64**, o1355. 10.1107/S1600536808018795 PMC2961731 21202974

[bb9] Obniska, J., Rzepka, S. & Kamiński, K. (2012). *Bioorg. Med. Chem.* **20**, 4872–4880. 10.1016/j.bmc.2012.05.032 22717240

[bb10] Sheldrick, G. M. (2008). *Acta Cryst.* A**64**, 112–122. 10.1107/S0108767307043930 18156677

[bb11] Westrip, S. P. (2010). *J. Appl. Cryst.* **43**, 920–925.

